# 2-[(*E*)-1-(4-Meth­oxy­phen­yl)pent-1-en-3-yl­idene]malononitrile

**DOI:** 10.1107/S1600536810045381

**Published:** 2010-11-13

**Authors:** Lian-Mei Chen, Tai-Ran Kang

**Affiliations:** a College of Chemistry and Chemical Engineering, China West Normal University, Nanchong 637002, People’s Republic of China

## Abstract

In the title compound, C_15_H_14_N_2_O, the mol­ecule skeleton displays an approximately planar structure except for the ethyl group [maximum deviation = 0.042 (1) Å]. The meth­oxy­phenyl ring and butanylidenemalononitrile groups are located on opposite sides of the C=C bond, showing an *E* configuration. Weak inter­molecular C—H⋯N hydrogen bonding is present in the crystal structure.

## Related literature

For the use of malononitrile-containing compounds as building blocks in synthesis, see: Liu *et al.* (2002 ▶); Sepiol & Milart (1985 ▶); Zhang *et al.* (2003 ▶). For a related structure, see: Kang & Chen (2009 ▶).
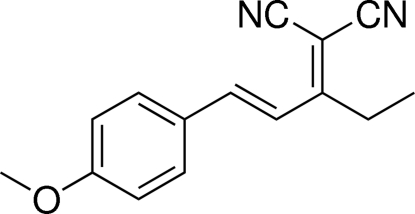


## Experimental

### 

#### Crystal data

C_15_H_14_N_2_O
*M* *_r_* = 238.28
Monoclinic, 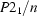

*a* = 12.3371 (3) Å
*b* = 8.8832 (2) Å
*c* = 12.8554 (3) Å
β = 108.050 (2)°
*V* = 1339.53 (5) Å^3^
*Z* = 4
Cu *K*α radiation
μ = 0.60 mm^−1^
*T* = 291 K
0.42 × 0.40 × 0.36 mm

#### Data collection

Oxford Diffraction Gemini S Ultra diffractometer
Absorption correction: multi-scan (*CrysAlis PRO*; Oxford Diffraction, 2009 ▶) *T*_min_ = 0.787, *T*_max_ = 0.813
9020 measured reflections
2456 independent reflections
2266 reflections with *I* > 2σ(*I*)
*R*_int_ = 0.014

#### Refinement

*R*[*F* ^2^ > 2σ(*F* ^2^)] = 0.039
*wR*(*F* ^2^) = 0.112
*S* = 1.05
2456 reflections
165 parameters
3 restraints
H-atom parameters constrained
Δρ_max_ = 0.11 e Å^−3^
Δρ_min_ = −0.14 e Å^−3^

### 

Data collection: *CrysAlis CCD* (Oxford Diffraction, 2008 ▶); cell refinement: *CrysAlis RED* (Oxford Diffraction, 2008 ▶); data reduction: *CrysAlis RED*; program(s) used to solve structure: *SHELXS97* (Sheldrick, 2008 ▶); program(s) used to refine structure: *SHELXL97* (Sheldrick, 2008 ▶); molecular graphics: *ORTEP-3* (Farrugia, 1997 ▶); software used to prepare material for publication: *SHELXL97*.

## Supplementary Material

Crystal structure: contains datablocks global, I. DOI: 10.1107/S1600536810045381/xu5082sup1.cif

Structure factors: contains datablocks I. DOI: 10.1107/S1600536810045381/xu5082Isup2.hkl

Additional supplementary materials:  crystallographic information; 3D view; checkCIF report

## Figures and Tables

**Table 1:** Hydrogen-bond geometry (Å, °)

*D*—H⋯*A*	*D*—H	H⋯*A*	*D*⋯*A*	*D*—H⋯*A*
C2—H2⋯N1^i^	0.93	2.62	3.5285 (19)	167 (1)

Symmetry code: (i) 
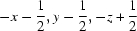
.

**Fig. 1.**
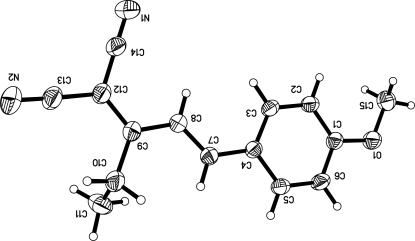
The molecular structure of (I) with 30% probability displacement ellipsoids (arbitrary spheres for H atoms).

## supplementary crystallographic information

### Comment

The chemistry of ylidene malononitrile have been studied extensively, from the ring closure reactions, the comounds containing newly formed five or six-membered rings, such as indans (Zhang *et al.*2003), naphthalenes (Liu, *et al.*2002), benzenes (Sepiol *et al.*1985) were obtained. Some crystal structures involving ylidene malononitrile groups have been published, including a recent report from our labratory (Kang *et al.*, 2009). As a part of our interest in the synthsis of some complex ring systems, we investigated the title compound (I), which is a diene reagent in Diels-Alder reaction. We report herein the crystal structure of the title compound.

The molecular structure of (I) is shown in Fig. 1. Bond lengths and angles in (I) are normal. The phenyl ring with two double bond and triple bond is co-planar. The crystal packing is stabilized by C—H···N hydrogen bonding (Table 1).

### Experimental

2-(Butan-2-ylidene)malononitrile (0.24 g 2 mmol) and 4-methoxy -benzaldehyde (0.272 g 2 mmol) were dissolved in 2-propanol (2 ml). To the solution was added piperidine (0.017 g, 0.2 mmol), the solution was stirred for 24 h at 343 K. Then the solution was cooled to room temperature, and was filtered to obtain a yellow solid. Recrystallization from hot ethanol afforded the pure compound. Single crystals of (I) suitable for X-ray analysis were obtained by slow evaporation ethanol solvent.

### Refinement

The carbon-bound hydrogen atoms were placed in calculated positions, with C—H = 0.93–0.97 Å, and refined using a riding model, with *U*_iso_(H) =1.5*U*_eq_(C) for methyl H atoms and *U*_iso_(H) =1.2*U*_eq_(C) for the others.

### Crystal data

**Table:**

C_15_H_14_N_2_O	*F*(000) = 504
*M**_r_* = 238.28	*D*_x_ = 1.182 Mg m^−^^3^
Monoclinic, *P*2_1_/*n*	Cu *K*α radiation, λ = 1.54184 Å
Hall symbol: -P 2yn	Cell parameters from 6882 reflections
*a* = 12.3371 (3) Å	θ = 3.6–69.4°
*b* = 8.8832 (2) Å	µ = 0.60 mm^−^^1^
*c* = 12.8554 (3) Å	*T* = 291 K
β = 108.050 (2)°	Block, yellow
*V* = 1339.53 (5) Å^3^	0.42 × 0.40 × 0.36 mm
*Z* = 4	

### Data collection

**Table:**

Oxford Diffraction Gemini S Ultra diffractometer	2456 independent reflections
Radiation source: Enhance Ultra (Cu) X-ray Source	2266 reflections with *I* > 2σ(*I*)
mirror	*R*_int_ = 0.014
Detector resolution: 15.9149 pixels mm^-1^	θ_max_ = 69.6°, θ_min_ = 4.3°
ω scans	*h* = −14→14
Absorption correction: multi-scan (*CrysAlis PRO*; Oxford Diffraction, 2009)	*k* = −10→10
*T*_min_ = 0.787, *T*_max_ = 0.813	*l* = −15→8
9020 measured reflections	

### Refinement

**Table:**

Refinement on *F*^2^	Primary atom site location: structure-invariant direct methods
Least-squares matrix: full	Secondary atom site location: difference Fourier map
*R*[*F*^2^ > 2σ(*F*^2^)] = 0.039	Hydrogen site location: inferred from neighbouring sites
*wR*(*F*^2^) = 0.112	H-atom parameters constrained
*S* = 1.05	*w* = 1/[σ^2^(*F*_o_^2^) + (0.0607*P*)^2^ + 0.1456*P*] where *P* = (*F*_o_^2^ + 2*F*_c_^2^)/3
2456 reflections	(Δ/σ)_max_ < 0.001
165 parameters	Δρ_max_ = 0.11 e Å^−^^3^
3 restraints	Δρ_min_ = −0.14 e Å^−^^3^

### Special details

**Table:**

Geometry. All e.s.d.'s (except the e.s.d. in the dihedral angle between two l.s. planes) are estimated using the full covariance matrix. The cell e.s.d.'s are taken into account individually in the estimation of e.s.d.'s in distances, angles and torsion angles; correlations between e.s.d.'s in cell parameters are only used when they are defined by crystal symmetry. An approximate (isotropic) treatment of cell e.s.d.'s is used for estimating e.s.d.'s involving l.s. planes.
Refinement. Refinement of *F*^2^ against ALL reflections. The weighted *R*-factor *wR* and goodness of fit *S* are based on *F*^2^, conventional *R*-factors *R* are based on *F*, with *F* set to zero for negative *F*^2^. The threshold expression of *F*^2^ > σ(*F*^2^) is used only for calculating *R*-factors(gt) *etc*. and is not relevant to the choice of reflections for refinement. *R*-factors based on *F*^2^ are statistically about twice as large as those based on *F*, and *R*- factors based on ALL data will be even larger.

### Fractional atomic coordinates and isotropic or equivalent isotropic displacement parameters (Å^2^)

**Table:**

	*x*	*y*	*z*	*U*_iso_*/*U*_eq_	
O1	0.09486 (7)	−0.22305 (10)	0.03379 (7)	0.0668 (3)	
C14	−0.13644 (11)	0.27243 (16)	0.51588 (10)	0.0646 (3)	
C7	0.13090 (9)	0.13033 (12)	0.40443 (8)	0.0486 (3)	
H7	0.2053	0.1581	0.4428	0.058*	
C2	−0.00061 (9)	−0.10045 (13)	0.15020 (9)	0.0534 (3)	
H2	−0.0720	−0.1348	0.1083	0.064*	
C8	0.04916 (9)	0.17842 (13)	0.44506 (8)	0.0505 (3)	
H8	−0.0254	0.1492	0.4090	0.061*	
C4	0.11576 (9)	0.03969 (11)	0.30705 (8)	0.0464 (3)	
C5	0.21124 (9)	0.00232 (14)	0.27569 (10)	0.0559 (3)	
H5	0.2828	0.0364	0.3174	0.067*	
C1	0.09578 (9)	−0.13673 (12)	0.12143 (9)	0.0512 (3)	
C3	0.01045 (9)	−0.01297 (13)	0.24151 (9)	0.0525 (3)	
H3	−0.0546	0.0117	0.2599	0.063*	
C6	0.20146 (9)	−0.08369 (14)	0.18453 (10)	0.0611 (3)	
H6	0.2661	−0.1066	0.1649	0.073*	
C9	0.06860 (9)	0.27216 (12)	0.54059 (9)	0.0507 (3)	
N1	−0.22774 (11)	0.23824 (19)	0.46897 (11)	0.0962 (5)	
C12	−0.02183 (10)	0.31596 (13)	0.57378 (9)	0.0552 (3)	
N2	−0.00312 (13)	0.48196 (17)	0.74197 (12)	0.0955 (4)	
C10	0.18745 (10)	0.31909 (15)	0.60535 (10)	0.0640 (3)	
H10A	0.1837	0.4087	0.6472	0.077*	
H10B	0.2297	0.3441	0.5554	0.077*	
C15	−0.01196 (12)	−0.28321 (15)	−0.03201 (11)	0.0687 (4)	
H15A	−0.0445	−0.3435	0.0128	0.103*	
H15B	−0.0002	−0.3444	−0.0891	0.103*	
H15C	−0.0629	−0.2022	−0.0638	0.103*	
C13	−0.00959 (11)	0.40823 (15)	0.66777 (11)	0.0670 (3)	
C11	0.25010 (13)	0.1964 (2)	0.68261 (12)	0.0890 (5)	
H11A	0.2143	0.1807	0.7382	0.133*	
H11B	0.3280	0.2260	0.7161	0.133*	
H11C	0.2477	0.1047	0.6424	0.133*	

### Atomic displacement parameters (Å^2^)

**Table:**

	*U*^11^	*U*^22^	*U*^33^	*U*^12^	*U*^13^	*U*^23^
O1	0.0564 (5)	0.0802 (6)	0.0658 (5)	0.0005 (4)	0.0219 (4)	−0.0264 (4)
C14	0.0545 (6)	0.0853 (9)	0.0560 (7)	0.0201 (5)	0.0199 (5)	0.0060 (5)
C7	0.0477 (5)	0.0515 (6)	0.0483 (5)	−0.0039 (4)	0.0173 (4)	−0.0007 (4)
C2	0.0417 (5)	0.0614 (6)	0.0569 (6)	−0.0034 (5)	0.0150 (4)	−0.0094 (5)
C8	0.0483 (6)	0.0564 (6)	0.0481 (6)	0.0002 (4)	0.0167 (4)	−0.0022 (5)
C4	0.0451 (5)	0.0490 (5)	0.0476 (5)	−0.0006 (4)	0.0179 (4)	0.0000 (4)
C5	0.0405 (5)	0.0666 (7)	0.0609 (6)	−0.0030 (5)	0.0163 (5)	−0.0113 (5)
C1	0.0505 (6)	0.0527 (6)	0.0526 (6)	0.0020 (5)	0.0194 (5)	−0.0070 (5)
C3	0.0426 (5)	0.0620 (6)	0.0576 (6)	−0.0014 (5)	0.0225 (5)	−0.0076 (5)
C6	0.0444 (6)	0.0742 (8)	0.0697 (7)	0.0008 (5)	0.0250 (5)	−0.0176 (6)
C9	0.0556 (6)	0.0511 (6)	0.0487 (6)	0.0024 (4)	0.0209 (5)	0.0016 (4)
N1	0.0536 (7)	0.1471 (13)	0.0840 (8)	0.0179 (7)	0.0154 (6)	0.0044 (8)
C12	0.0588 (6)	0.0598 (6)	0.0503 (6)	0.0116 (5)	0.0215 (5)	0.0034 (4)
N2	0.1005 (10)	0.1055 (10)	0.0906 (9)	0.0068 (8)	0.0443 (8)	−0.0336 (8)
C10	0.0619 (7)	0.0708 (7)	0.0652 (7)	−0.0132 (6)	0.0284 (6)	−0.0197 (6)
C15	0.0699 (8)	0.0710 (8)	0.0633 (7)	−0.0060 (6)	0.0179 (6)	−0.0210 (6)
C13	0.0705 (8)	0.0706 (8)	0.0664 (7)	0.0122 (6)	0.0308 (6)	−0.0064 (6)
C11	0.0617 (8)	0.1201 (13)	0.0720 (9)	−0.0096 (8)	0.0015 (7)	−0.0004 (9)

### Geometric parameters (Å, °)

**Table:**

O1—C1	1.3600 (13)	C1—C6	1.3873 (15)
O1—C15	1.4308 (15)	C3—H3	0.9300
C14—N1	1.1417 (18)	C6—H6	0.9300
C14—C12	1.4321 (18)	C9—C12	1.3686 (15)
C7—C8	1.3412 (15)	C9—C10	1.5035 (16)
C7—C4	1.4510 (14)	C12—C13	1.4289 (16)
C7—H7	0.9300	N2—C13	1.1393 (17)
C2—C3	1.3788 (15)	C10—C11	1.516 (2)
C2—C1	1.3883 (14)	C10—H10A	0.9700
C2—H2	0.9300	C10—H10B	0.9700
C8—C9	1.4410 (15)	C15—H15A	0.9600
C8—H8	0.9300	C15—H15B	0.9600
C4—C3	1.3923 (15)	C15—H15C	0.9600
C4—C5	1.3980 (15)	C11—H11A	0.9600
C5—C6	1.3727 (16)	C11—H11B	0.9600
C5—H5	0.9300	C11—H11C	0.9600
			
C1—O1—C15	118.04 (9)	C12—C9—C8	119.57 (10)
N1—C14—C12	179.45 (15)	C12—C9—C10	119.86 (10)
C8—C7—C4	126.97 (10)	C8—C9—C10	120.56 (9)
C8—C7—H7	116.5	C9—C12—C13	122.96 (11)
C4—C7—H7	116.5	C9—C12—C14	122.01 (11)
C3—C2—C1	119.38 (10)	C13—C12—C14	115.03 (10)
C3—C2—H2	120.3	C9—C10—C11	112.04 (11)
C1—C2—H2	120.3	C9—C10—H10A	109.2
C7—C8—C9	124.70 (10)	C11—C10—H10A	109.2
C7—C8—H8	117.7	C9—C10—H10B	109.2
C9—C8—H8	117.7	C11—C10—H10B	109.2
C3—C4—C5	117.22 (10)	H10A—C10—H10B	107.9
C3—C4—C7	123.72 (9)	O1—C15—H15A	109.5
C5—C4—C7	119.06 (10)	O1—C15—H15B	109.5
C6—C5—C4	121.29 (10)	H15A—C15—H15B	109.5
C6—C5—H5	119.4	O1—C15—H15C	109.5
C4—C5—H5	119.4	H15A—C15—H15C	109.5
O1—C1—C6	116.17 (9)	H15B—C15—H15C	109.5
O1—C1—C2	124.31 (10)	N2—C13—C12	178.06 (14)
C6—C1—C2	119.53 (10)	C10—C11—H11A	109.5
C2—C3—C4	122.15 (9)	C10—C11—H11B	109.5
C2—C3—H3	118.9	H11A—C11—H11B	109.5
C4—C3—H3	118.9	C10—C11—H11C	109.5
C5—C6—C1	120.42 (10)	H11A—C11—H11C	109.5
C5—C6—H6	119.8	H11B—C11—H11C	109.5
C1—C6—H6	119.8		
			
C4—C7—C8—C9	−178.09 (10)	C4—C5—C6—C1	−0.5 (2)
C8—C7—C4—C3	−1.00 (18)	O1—C1—C6—C5	−178.94 (11)
C8—C7—C4—C5	178.78 (11)	C2—C1—C6—C5	0.91 (19)
C3—C4—C5—C6	−0.49 (18)	C7—C8—C9—C12	179.18 (11)
C7—C4—C5—C6	179.72 (10)	C7—C8—C9—C10	−2.24 (17)
C15—O1—C1—C6	178.49 (11)	C8—C9—C12—C13	179.74 (10)
C15—O1—C1—C2	−1.35 (17)	C10—C9—C12—C13	1.14 (18)
C3—C2—C1—O1	179.44 (10)	C8—C9—C12—C14	−0.48 (18)
C3—C2—C1—C6	−0.40 (18)	C10—C9—C12—C14	−179.08 (11)
C1—C2—C3—C4	−0.58 (18)	C12—C9—C10—C11	98.07 (14)
C5—C4—C3—C2	1.01 (17)	C8—C9—C10—C11	−80.51 (14)
C7—C4—C3—C2	−179.20 (10)		

### Hydrogen-bond geometry (Å, °)

**Table:**

*D*—H···*A*	*D*—H	H···*A*	*D*···*A*	*D*—H···*A*
C2—H2···N1^i^	0.93	2.62	3.5285 (19)	167 (1)

Symmetry codes: (i) −*x*−1/2, *y*−1/2, −*z*+1/2.
